# Antibacterial Films of Alginate-CoNi-Coated Cellulose Paper Stabilized Co NPs for Dyes and Nitrophenol Degradation

**DOI:** 10.3390/polym13234122

**Published:** 2021-11-26

**Authors:** Yasir Anwar, Hani S. H. Mohammed Ali, Waseeq Ur Rehman, Hassan A. Hemeg, Shahid Ali Khan

**Affiliations:** 1 Department of Biological Sciences, Faculty of Science, King Abdulaziz University, P.O. Box 80203, Jeddah 21589, Saudi Arabia; haniolfat@gmail.com; 2 Princess Dr. Najla Bint Saud Al-Saud Center for Excellence Research in Biotechnology, King Abdulaziz University, P.O. Box 80203, Jeddah 21589, Saudi Arabia; 3 Department of Chemistry, Government Post Graduate College Nowshera, Nowshera 24100, Pakistan; gwaseeq@gmail.com; 4 Department of Medical Laboratory Technology, College of Applied Medical Sciences, Taibah University, Almadina Almunawra 30001, Saudi Arabia; hasanhemeg@hotmail.com; 5 Center of Excellence for Advanced Materials Research (CEAMR), King Abdulaziz University, P.O. Box 80203, Jeddah 21589, Saudi Arabia; 6 Department of Chemistry, University of Swabi, Swabi Anbar, Swabi 23561, Pakistan

**Keywords:** alginate, CoNi nanocomposite, cellulose paper, antibacterial potential, degradation

## Abstract

The development of a solid substrate for the support and stabilization of zero-valent metal nanoparticles (NPs) is the heart of the catalyst system. In the current embodiment, we have prepared solid support comprise of alginate-coated cellulose filter paper (Alg/FP) for the synthesis and stabilization of Co nanoparticles (NPs) named as Alg/FP@Co NPs. Furthermore, Alginate polymer was blended with 1 and 2 weight percent of CoNi NPs to make Alg-CoNi1/FP and Alg-CoNi2/FP, respectively. All these stabilizing matrixes were used as dip-catalyst for the degradation of azo dyes and reduction of 4-nitrophenol (4NP). The effect of initial dye concentration, amount of NaBH_4_, and catalyst dosage was assessed for the degradation of Congo red (CR) dye by using Alg-CoNi2/FP@Co NPs. Results indicated that the highest *k*_app_ value (3.63 × 10^−1^ min^−1^) was exhibited by Alg-CoNi2/FP@Co NPs and lowest by Alg/FP@Co NPs against the discoloration of CR dye. Furthermore, it was concluded that Alg-CoNi2/FP@Co NPs exhibited strong catalyst activity against CR, and methyl orange dye (MO) degradation as well as 4NP reduction. Antibacterial activity of the prepared composites was also investigated and the highest l activity was shown by Alg-CoNi2/FP@Co NPs, which inhibit 2.5 cm zone of bacteria compared to other catalysts.

## 1. Introduction

Nanoscience received much interest in recent years due to their vast applications in sensing, drug delivery, antimicrobial activity, coating, bio-imaging, environmental remediation, and biomedical applications [1,2,3,4,5,6]. Besides their major applications in various technological sectors, they are used as catalysts in many industrial and chemical reactions like hydrogen production [7], oxygen evolution reaction, organic reactions [8], and nitroaromatics reduction [9,10,11,12]. The above diverse applications of metallic nanoparticles are because of their high surface to volume ratio, high surface energy, and small quantum size effect [13]. The quantum size effect gives an amazing reduction in the particle size of the nanoparticles beyond the threshold limit and leads to the change in the electronic environment of the particle. Owing to the change in the electronic environment, small particle size, high surface area, and high surface energy lead to an abrupt increase in particle activity. However, due to the high energy associated with the particle, there exist some drawbacks with the usage of these particles as the catalyst. For instance, aggregation and agglomeration of the particles are due to the physical entanglement and Wander Waal’s forces [14]. The second most common problem in nanoparticles research is their separation after the reaction completion. Both the problems can be overcome by the use of a solid matrix that not only avoids particle aggregation, but also is easily separated from the reaction mixture. The separation of a catalyst by an easy method from the reaction mixture at any time is called a dip-catalyst. Moreover, the use of a solid support improves the stability of the nanoparticles. For example, using solid support not only avoids aggregation, but also prevents the leaching of nanoparticles, which otherwise cause’s environmental issues. Many solid supports have been used for the stabilization of nanoparticles such as carbon [15], metal oxide [16], inorganic (e.g., silica (Li et al., 2013)), or organic materials [17]. Organic solid support is the most frequently used solid support for this purpose due to its inert nature to the reaction and ease of derivatization by introducing new functionality to the host material. However, among all these supporting materials, polymer-supported substrate for nanoparticles stabilization is one of the most important systems. For instance, Pd nanoparticles have been supported on polymethyl methacrylate spheres [18], and fabricated polystyrene microspheres supported Pt nanoparticles are examples of this [19]. Some approaches have been applied to use cellulose polymer as solid support for various metal nanoparticles. For example, some researchers have used cellulose cotton in the form of filter paper for metallic nanoparticle stabilization [20]. Cellulose is one of the most abundant natural organic polymers and is used in composite and packaging materials, and are also used in textile industries. The *β* 1,4 glycoside linked the *β*-d-anhydroglucopyranose monomers through one to four linkages. This linear cellulose molecule has three –OH groups in its monomer unit, which are responsible for the stabilization of metal nanoparticles. Furthermore, the cellulose polymer-supported NPs act as a dip-catalyst, and one can remove the catalyst from the reaction medium easily [21]. Although, cellulose microfibers have multiple –OH groups in their polymer network, but low process-ability, it was not considered for using directly as a stabilizing matrix for Co NPs. Furthermore, the alginate polymer swells easily and demonstrated a strong gelling characteristics to make hydrogels. Therefore, both cellulose microfibers and alginate polymer provide a good platform for the stabilization of NPs. Moreover, the use of alginate biopolymer to coat the surface of cellulose paper has markedly enhanced the active functionalities that further stabilize the metal nanoparticles. For instance, the presence of –OH and –COOH groups in the alginate biopolymer further increases the stabilization of the Co NPs [22,23,24]. It is reported that inorganic polymer hybrid material greatly enhanced the properties of the materials [25]. Therefore, CoNi nanocomposite was incorporated in the alginate polymer host material to achieve a hybrid composite.

Mostly the supported nanoparticles were used for the degradation of organic pollutants in an aqueous medium. Nitrophenols and azo dyes are among the most noticeable organic pollutants. The two common nitrophenols are 2- and 4-nitrophenols and various industries produce them in a large amount. If an industry produces one nitrophenol, the other will be formed in small quantities and vice versa. Both nitrophenols find large applications in pharmaceutical, agriculture, fabric, dye, cosmetic, and explosive industries. Nitrophenols are health hazards and many health issues are associated with them, for instance, high exposure to them can irritate eyes, nose, throat, skin, and possibly damage the eye. Furthermore, exposure to 4-nitrophenols can cause stomach disorders, enhance heartbeat, and may affect the central nervous system [26,27]. 

Furthermore, azo dyes contributed two-thirds of all the synthetic dyes and are used in large amounts in the pharmaceutical industry [28,29,30]. They are used in the pharmaceutical industry to color various drugs; however, carcinogenic and mutagenic activities of many azo dyes are reported in the literature. Azo dyes also find applications in the fabric and cosmetics industries. Moreover, the carcinogenic effect of most azo dyes depends on the structure of dye molecule and the way they are degraded [31,32].

In the current study, we have prepared alginate-based cellulose filter paper (FP) as solid support for t zero-valent Co NPs and used it in the degradation of CR and MO dyes as well as 4NP in the presence of borohydride in an aqueous medium.

## 2. Experimental Section

### 2.1. Reagents and Materials

Congo red, (CAS no. 573-58-0) methyl orange, (CAS no. 547-58-0), and NaBH_4_ (CAS no. 16940-66-2) were purchased from DAEJUNG Company (Sasang-gu, Busan, Korea). Similarly, other chemicals such as, NaOH, CoCl_2_·6H_2_O, and NiCl_2_·6H_2_O were acquired from Sigma Aldrich (Kawasaki, Kanagawa, Japan), and deionized water was used throughout the experiment. Sodium Alginate polymer (CAS no. 9005-38-3) was purchased from DAEJUNG Company (Sasang-gu, Busan, Korea), while Whatman filter paper was used as a source of cellulose paper and was obtained from Sigma Aldrich (Kawasaki, Kanagawa, Japan).

### 2.2. Synthesis of Catalyst

#### 2.2.1. Synthesis of CoNi Nanocomposite

CoNi nanocomposite was synthesized via the sol-gel method using the previous procedure [20,21,22]. An equal amount of 0.5 mM solution of CoCl_2_·6H_2_O, and NiCl_2_·6H_2_O was mixed in the stoichiometric ratio in a 1 L flask and mixed for 30 min. After that, a dilute solution of NaOH was added to the solution till pH 10, and heated for 7 h at 70 °C with uniform magnetic stirring. The product formed was washed with a 1:4 ethanol-water mixture, and heated in an oven for 8 h at 50 °C. The dried product was calcined and activated at 400 °C for 6 h. 

#### 2.2.2. Preparation of Alg/FP

The alginate polymer (2 g) was thoroughly dispersed in 50 mL distilled water through constant stirring to make a viscous solution. After that, 5 pieces (2 × 6 cm^2^) of Whatman filter paper (FP) were put in the alginate solution for 6 h to coat the FP and then dried at room temperature. 

#### 2.2.3. Preparation of Alg-CoNi1/FP and Alg-CoNi2/FP

Both Alg-CoNi1/FP and Alg-CoNi2/FP were synthesized in three steps.

In the first step, 2 g alginate polymer was dissolved in 50 mL distilled water in two separate beakers to make their viscous solutions. In the second step, 1 and 2 weight% of CoNi composite was dispersed in each separate beaker and homogenized through continuous stirring, respectively. In the third step, the FP was placed in the above mixture for 6 h and then dried at room temperature. 

#### 2.2.4. Preparation of Alg-CoNi1/FP@Co and Alg-CoNi2/FP@Co NPs

**Scheme 1:**
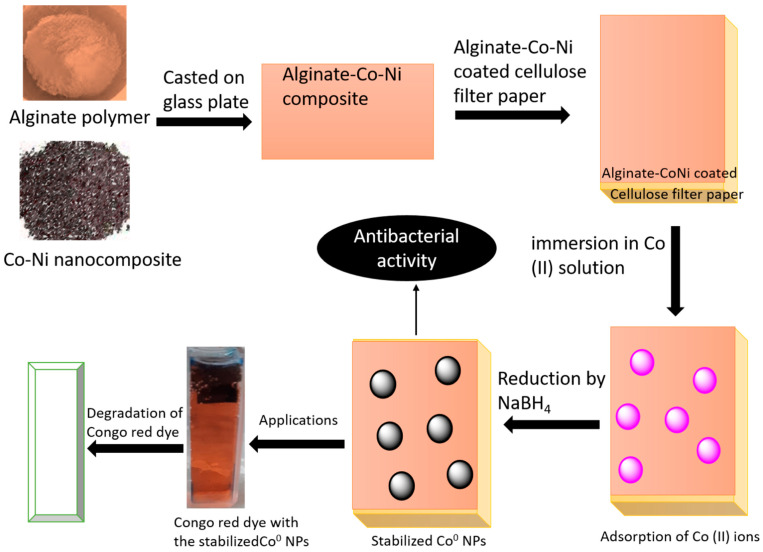
Pictorial representation of the preparation of Alg-CoNi/FP@Co NPs and their applications against bacterial inhibition and degradation of dyes.

The Alg-CoNi1/FP and Alg-CoNi2/FP substrate were placed in 0.5 mM Co (II) salt solution for 6 h and then rinsed with distilled water and dried. The dried Alg-CoNi1/FP and Alg-CoNi2/FP adsorbed Co (II) ions were dipped in 0.1 mM NaBH_4_ fresh solution for 5 min, which converted Co ions to zero-valent Co NPs (Co^0^). The as-synthesized Co NPs were directly used for the antibacterial studies and degradation of azo dyes or 4NP reduction as shown in Scheme 1.

The following equation indicated the conversion of Co (II) ions to their Co^0^ NPs [33].

Co^+2^ + BH_4_^−^ + 3H_2_O + 2e^−^ → Co^0^ + H_3_BO_3_ + 7H^+^

3Co^0^ + 6H^+^ → 3Co^+2^ + 3H_2_

Both Co^+2^ and Co^0^ were supported on Alg/FP, Alg-CoNi1/FP, and Alg-CoNi2/FP solid substrate.

### 2.3. Antibacterial Activity

The antibacterial potential of Alg/FP, Alg/FP@Co, Alg-CoNi1/FP@Co, and Alg-CoNi2/FP@Co NPs was evaluated on a muller Hinton agar plates against *B. subtilis*. The antibacterial method was based on the Kirby−Bauer disk diffusion method with minor changes in the procedure [34]. The plates were prepared, sterilized, solidified, and then the culture of *B. subtilis* was spread thoroughly via a sterilized spreader on the whole plate. After that, each catalyst was cut in a specified dimension and then placed in the bacterial zone. The plates were placed at 37 °C for 24 h. After incubation, the zone of inhibition was measured. The experiments were performed in triplicate and the performance of the catalysts was determined by calculating the mean zone of inhibition around the nutrient agar disk. 

### 2.4. Pollutants Degradation Experiment

All three catalysts were applied for the degradation of azo dyes and reduction of 4NP. The concentration of both dyes was 0.07 mM. In the batch experiment, 3 mL of each dye solution was put in a quartz cuvette with the addition of 0.5 mL of NaBH_4_ solution and 25 mg of each catalyst. After that, the reaction was constantly monitored through UV-Vis spectrophotometer with a 1 min interval time. The decrease in absorbance at 495 and 464 nm for CR and MO dyes were recorded, respectively, until complete discoloration. The effect of initial dye concentration, NaBH_4_ amount, and catalyst dosage was evaluated on the degradation of CR dye. 

The degradation of dyes or nitrophenol in percent can be deduced from the following Equation (1).

(1) $$\%\;\mathrm{reduction}/\mathrm{degradation}=\frac{\left(C_{0}-C_{t}\right)}{C_{0}}\times 100$$

where C_0_ and C_t_ represent the initial and final concentrations of the solution after passing time t.

While the rate constant *k*_app_ was deduced from the linear relationship of lnC_t_/C_0_ vs. t as shown in Equation (2).

(2) $$\ln\frac{C_{t}}{C_{0}}=-\mathrm{kt}\;+C$$

### 2.5. Physiochemical Characterization

The crystalline nature of the catalyst was performed on X-ray diffraction technique (HighTech) with Cu K*α* radiations source (λ = 0.154 nm) having 25 mA current and 40 kV voltage, while the scan range was recorded from 15–80 nm with step time 1 s and step size 0.05 degrees. FTIR was recorded on Autonuated total reflectance-Fourier transformed infrared spectroscopy machine of Thermo scientific Company from 400–4000 cm^−1^ range and FESEM on JEOL (JSM-7600F, Tokyo, Japan) and EDS (EDS oxford system, Oxford, UK). UV-Vis spectrophotometer of Thermo Scientific Evolution Company was used for the catalytic degradation experiment.

## 3. Results and Discussion

### 3.1. Characterizations

#### 3.1.1. FESEM

**Figure 1:**
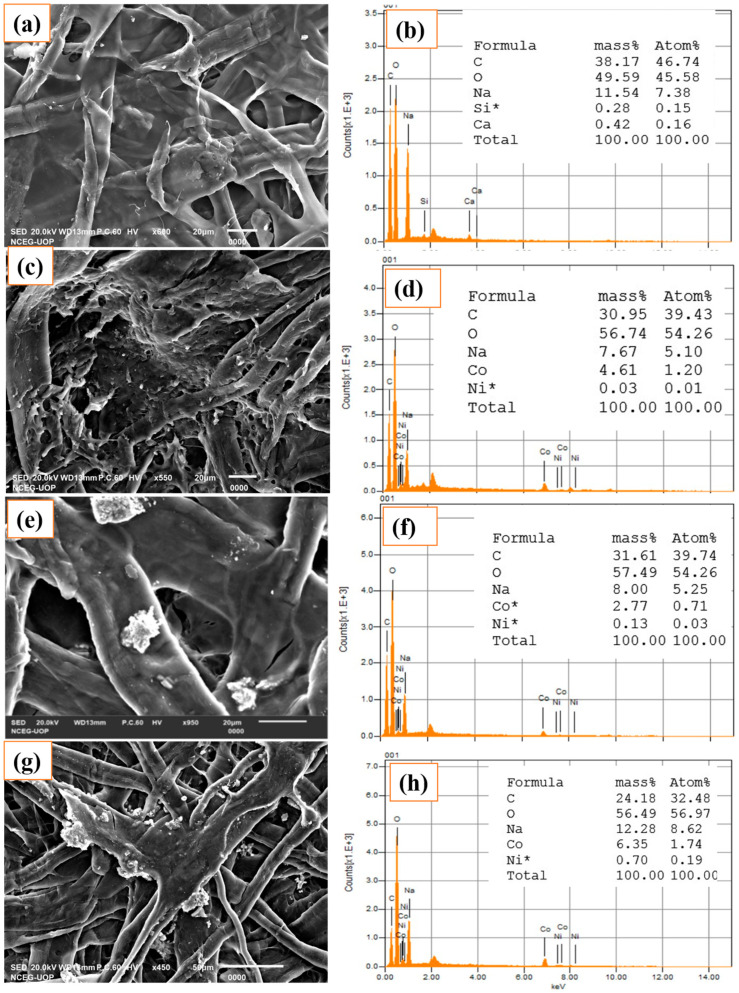
The left-hand side are the FESEM images that give the right-hand side elemental window with their mass and atomic percentage. The FESEM and EDS images of Alg/FP (**a**,**b**), Alg/FP@Co NPs (**c**,**d**), Alg-CoNi1/FP@Co (**e**,**f**), and Alg-CoNi2/FP@Co NPs (**g**,**h**), respectively. The star indicated in the EDS window are the Ni and Co. The EDS vertical axes indicate the scientific notation as 1 x 10^3^.

The FESEM images of Alg/FP indicated the presence of alginate polymer on the surface of cellulose microfibers. Fibers are covered by a thin layer of the alginate polymer as shown in the Figure 1a. The Alg/FP@Co NPs indicated small particles on the surface of the alginate layer as well as cellulose microfibers. These small spherical particles indicated the presence of Co NPs (Figure 1c). The Alg-CoNi1/FP@Co NPs also showed a similar display of the alginate polymer covering the cellulose microfiber, where the CoNi are protruded from the alginate layer. Furthermore, Co NPs are stabilized on Alg-CoNi1/FP layer (Figure 1e). The same explanation is true for Alg-CoNi2/FP@Co as discussed for the Alg-CoNi1/FP@Co NPs (Figure 1g).

#### 3.1.2. EDS

The EDS spectrum of all the catalysts is indicated in the inset of Figure 1. On the left side, the FESEM images of the catalyst are presented from which the right side elemental window are derived. For instance, Figure 1b indicated FESEM images and the EDS spectrum of Alg/FP, which indicated C and O as the main element along with Na, Ca, and Si. The C and O elements are 38.59 and 49.59 by mass%. Similarly, Alg/FP@Co NPs indicated C, O, and Co elements in 30.95, 56.74, and 4.61 by mass% (Figure 1d). The Alg-CoNi1/FP@Co NPs showing C, O, Ni, and Co elements in 31.61, 57.49, 0.13, and 2.77 by mass%, respectively (Figure 1f). Similarly, Alg-CoNi1/FP@Co NPs C, O, Ni, and Co elements in 24.18, 56.49, 0.70, and 6.35 by mass% respectively (Figure 1h). In all the elemental windows, Na has appeared in different ratios because the alginate polymer is used in the form of their sodium salt. 

#### 3.1.3. FTIR

**Figure 2:**
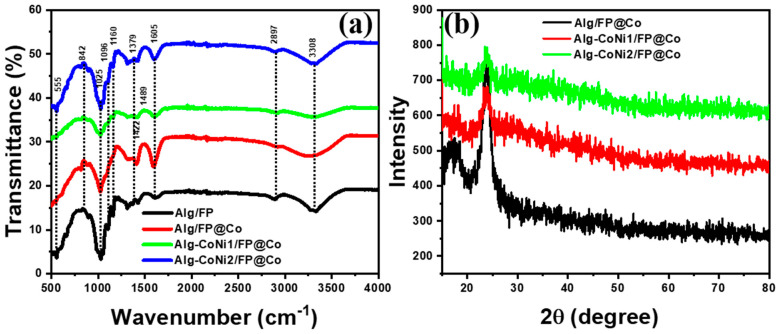
FTIR (**a**) and XRD (**b**) of Alg/FP, Alg-CoNi1/FP@Co, and Alg-CoNi2/FP@Co NPs.

The FTIR spectra revealed many peaks below 1600 cm^−1^ due to the presence of various functionalities of the polymers backbone. Similarly, there are two peaks above 1600 cm^−1^, which are displayed at 2897 and 3308 cm^−1^ due to the presence of C-H and O-H asymmetric stretching vibrations (Figure 2a). Furthermore, the broadness of peaks at 3308 cm^−1^ in all catalysts is due to the presence of multiple intra or inter-molecular H-binding in alginate and cellulose microfibers [21]. The absorbance at 1605 cm^−1^ suggests the adsorbed water on the polymer surface, while the acetate group exhibited peak at 1489 cm^−1^. The acetate groups are present in both cellulose microfibers and alginate polymer host material. Moreover, the characteristic polysaccharides peaks appeared in the range of 1422–555 cm^−1^. For instance, peaks at 1422, 1379, 1160, and 1096 cm^−1^ indicated the presence of –CH_2_, –CH_3_, and –OH and C-O bending vibrations [25]. Besides, the peak in the range of 1420–1430 cm^−1^ suggesting the crystalline nature of polysaccharide, while the amorphous amount appeared in the range of 842 cm^−1^ in the polymer backbone [35]. The absorbance peak at 555 cm^−1^ is the characteristic of the metal–oxygen bond [36], which is prominent in the Alg-CoNi2/FP@Co NPs. The fingerprint region also appeared in the range of 800–500 cm^−1^, therefore, this peak also exhibited in another catalyst. The above discussion inferred that the synthesized catalysts have a polymer backbone with little difference in the absorbance peaks. The small difference in the absorbance peaks among the catalyst peaks and from the literature data is due to the interconnectivity of alginate polymer with CoNi catalyst and cellulose microfibers. 

#### 3.1.4. XRD

XRD spectra indicated sharp crystalline peaks for cellulose microfibers at 2θ = 17.0° and 23.1°, which correspond to the crystal-I plane of cellulose microfibers at (110) and (200) planes, respectively (Figure 2b). The Co NPs did not appear in all the catalysts, suggesting their poor crystalline growth during the process at room temperature. Similar observations were also reported in the literature during the synthesis of Co and Cu NPs, respectively [37,38]. Therefore, we suggest that bigger particles of Co NPs formed due to aggregation.

### 3.2. Antibacterial Characteristics

Microbial contamination is a major issue in various technological fields such as the food industry, personal care products, water industry, medical devices, hospitals appliances, babies’ toys, hospital appliances, surgical apparatus, food and beverages packaging, textiles industry, and many other daily life usages [39,40,41]. The synthesis and applications of antimicrobial materials are exponentially increasing and researchers are trying to find new materials that can inhibit or kill the microbes. Recently, the advent of nanoscale materials finds its numerous applications against bacterial and microbial killing or inhibition. Therefore, these materials are largely studied at both industrial and academic levels because they provided substantial importance to other materials. Once the material is proved as an antimicrobial agent its use and application became extended to other technological sectors. In this study, we have screened the Alg/FP, Alg/FP@Co, Alg-CoNi1/FP@Co, and Alg-CoNi2/FP@Co catalysts against the inhibition of *B. subtilis* Gram-positive bacterium. The highest zone of inhibition of 2.5 cm was achieved with Alg-CoNi2/FP@Co NPs and lowest with Alg/FP, which suggested the role of CoNi NPs in Alginate-cellulose filter paper network. Ting Tsai et al. studied the antibacterial activity of cellulose paper fabricated with Ag-coated Au NPs indicating strong antibacterial activity against *E. coli* JM109. The authors synthesized Ag-coated Au NPs in different sizes, where the particles with 15 nm showed an excellent antibacterial activity against *E. coli* [42]. Similarly, bacterial cellulose fabricated with Ag NPs showed good antibacterial potential against *E. coli*. Thus, it is suggested that cellulose materials can be modified with various inorganic filler to make it an efficient catalyst against bacterial inhibition [43]. The zone of inhibition *B. subtilis* by all catalysts is provided in Table 1.

### 3.3. Catalyst activity

#### 3.3.1. Discoloration of CR Dye

**Figure 3:**
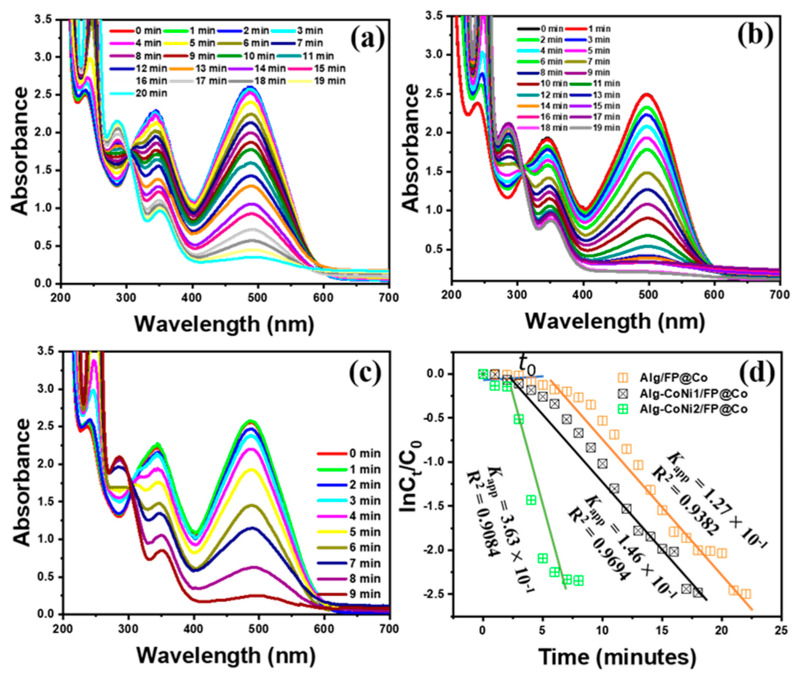
UV-Vis. absorbance spectra of CR dye discoloration in the presence of NaBH_4_ using Alg/FP@Co (**a**), Alg-CoNi1/FP@Co (**b**), Alg-CoNi2/FP@Co NPs (**c**), and linear relationship of pseudo-1st order kinetics (**d**).

The as-synthesized Co NPs on the Alg/FP and Alg-CoNi1/FP@Co NPs and AlgCoNi2/FP@Co NPs were used as solid matrix and were applied against the decolorization of CR dye. CR dye appeared at 495 nm in the UV-Vis. absorbance spectrum. This redshift is due to the presence of the diazo group, which upon treatment with NaBH_4_ transformed to hydrazine products [44]. A literature survey discovered that NaBH_4_ can decolorize CR dye, but the process is too slow and has no economic importance. The process is thermodynamically important, but not kinetically [44,45]. However, metal nanoparticles have proved to have an important effect on the degradation of CR dye. Alg/FP@Co, Alg-CoNi1/FP@Co, and Alg-CoNi2/FP@Co NPs were used against the degradation of CR dye in the presence of NaBH_4_ as a reducing agent. As discussed earlier, the NaBH_4_ has a negligible effect on the degradation of CR dye; however, it is thus required for the CR degradation along with the Co NPs to overcome the activation barrier of the reaction. Briefly, after the addition of the catalyst, there was a decline in the absorbance of CR dye at λ_max_ 495 nm, and some other peaks were arising at 344, 284, and 244 nm during the discoloration of CR dye. The appearance of new peaks predicted the formation of byproducts. These new peaks were due to the –COOH and –NH_2_ groups, respectively [44]. As manifested in the inset of Figure 3a, the Alg/FP supported Co NPs degraded the CR dye in 20 min with *k*_app_ value 1.27 × 10^−1^ min^−1^ with a regression coefficient R^2^ 0.9382. The degradation percent of CR was 91.76% per 20 min (Table 2). Similarly, under the same experimental conditions, 91.65 and 90.41% of the CR dye solution was decolorized in 19 and 9 min by Alg-CoNi1/FP@Co and Alg-CoNi2/FP@Co NPs, respectively. The rate constant values of CR discoloration with Alg-CoNi1/FP@Co and Alg-CoNi2/FP@Co NPs were 1.46 × 10^−1^, 3.63 × 10^−1^ min^−1^, respectively. Based on the *k*_app_ values, the superior catalyst activity was displayed by Alg-CoNi2/FP@Co NPs compared to Alg/FP@Co NPs and Alg-CoNi1/FP@Co NPs. The UV-Vis. absorbance spectrum of CR dye catalyzed by Alg-CoNi1/FP@Co and Alg-CoNi2/FP@Co NPs manifested in Figure 3b,c, respectively, while the linear relationship based on lnC_t_/C_0_ vs. time is depicted in Figure 3d. The slow rate of Alg/FP supported Co NPs against the degradation of CR dye was due to an induction period (*t*_0_), as shown in Figure 3d. This period is characterized by the rearrangement of reactive sites of the catalyst such as faces and edges. Once these reactive sites are prepared for the chemical reactions, the rate of reaction is enhanced. A high *t*_0_ value was observed for Alg/FP@Co NPs as compared to Alg/FP-Co-Ni1@Co NPs and Alg/FP-Co-Ni2@Co NPs, which further support the role of CoNi nanocatalyst in the Alg/FP polymer networks. It is further proposed that during the CR degradation, NaBH_4_ and CR dye get adsorbed on the surface of Co NPs supported on Alg/FP or Alg-CoNi/FP. After the adsorption of NaBH_4_ and CR dye, the Co NPs transferred the electrons provided by NaBH_4_ in the form of H^−1^ ions to the dyes. Thus, Co NPs supported on the solid matrix provided a vast surface for the CR dye, NaBH_4_, and electrons, where they play with each other and degraded the CR dye [46]. 

After concluding the high catalyst activity of Alg-CoNi/2FP@Co NPs, various factors such as effect of initial dye concentration, the effect of NaBH_4_ and catalyst amount were studied on the degradation of CR dye by using Alg-CoNi/2FP@Co NPs in the presence of NaBH_4_.

##### Effect of Concentration on CR Dye Degradation

Concentration has a major role in the degradation of pollutants because the reaction occurs on the surface of catalyst. It is well-known that increasing the amount of concentration will decrease the rate of reaction because high amount of pollutant molecules is available for the same amount of catalyst. Various concentrations of CR dye such as 0.03, 0.05, and 0.09 mM were studied by using 0.5 mL of NaBH_4_ and 30 mg of the Alg-CoNi/2FP@Co NPs (Table 3).

##### Effect of NaBH_4_ on the Discoloration of CR Dye

**Figure 4:**
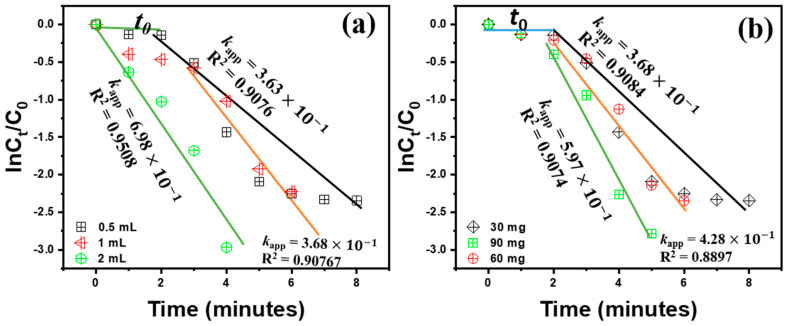
Pseudo first order kinetics plots of CR dye discoloration under the influence of different amount of NaBH_4_ (30 mg catalyst, 0.09 mM CR dye) (**a**) and catalyst amount (0.05 mL of 1 mM NaBH_4_ solution, 0.09 mM CR dye) (**b**).

The effect of NaBH_4_ was studied by changing the volume (0.5, 1, and 2 mL) of 1 mM NaBH_4_ solution while keeping the same concentration and volume of CR dye (0.09 mM in 3.5 mL solution), and 30 mg of the Alg-CoNi/2FP@Co NPs. Increasing the volume of NaBH_4_ solution from 0.5 to 2 mL, the rate of reaction also enhanced from 3.63 × 10^−1^, 3.68 × 10^−1^, and 6.98 × 10^−1^ min^−1^, respectively. This indicated that the rate of reaction can be enhanced with increase in volume of NaBH_4_, which suggests the prominent role of NaBH_4_ in the degradation of CR dye (Figure 4a and Table 3). According to the Hinshelwood-Langmuir mechanism, both BH_4_^−^ and reactant adsorbed on the surface of the NPs. Note that the adsorption of BH_4_^−^ and reactant is a reversible process, which makes competition among both BH_4_^¯^ and reactant for the active sites of the NPs. Therefore, a high concentration of reactant slows down the reaction rate. As is obvious from Table 3, the slow rate was observed with high CR concentration. Similarly, a high volume of NaBH_4_ increases the rate of reaction. For instance, a *k*_app_ 6.98 × 10^−1^ min^−1^ is observed when the amount of NaBH_4_ is high. Both CR and BH_4_^−^ accommodate on the surface of NPs in a reversible manner where the BH_4_^−^ provided surface hydrogen to the CR and convert it to the hydrazine derivatives. These hydrazine derivatives further degraded the product. After the reaction completion, the product detached from the NPs surface and made it free for the next cycle.

##### Effect of Catalyst Dosage on the Discoloration of CR Dye

Optimizing the catalyst amount for the discoloration of reactant is highly desirable in the field of nanocatalysis. Increasing amount of the catalyst will increase the active sites of the catalyst for the molecules. Therefore, the reactant and BH_4_^−^ accommodate easily on the surface of the catalyst. For instance, 30, 60, and 90 mg of the Alg-CoNi2/FP@Co NPs was used against CR dye degradation under the same experimental conditions. At 90 mg, the rate of reaction was *k*_app_ 5.97 × 10^−1^ min^−1^ compared to 3.68 × 10^−1^ min^−1^ with 30 mg of the catalyst. This suggested that at high amount of the catalyst, the rate of reaction is also high owing to the availability of more active sites for reaction (Table 3 and Figure 4b). 

#### 3.3.2. Discoloration of MO Dye

**Figure 5:**
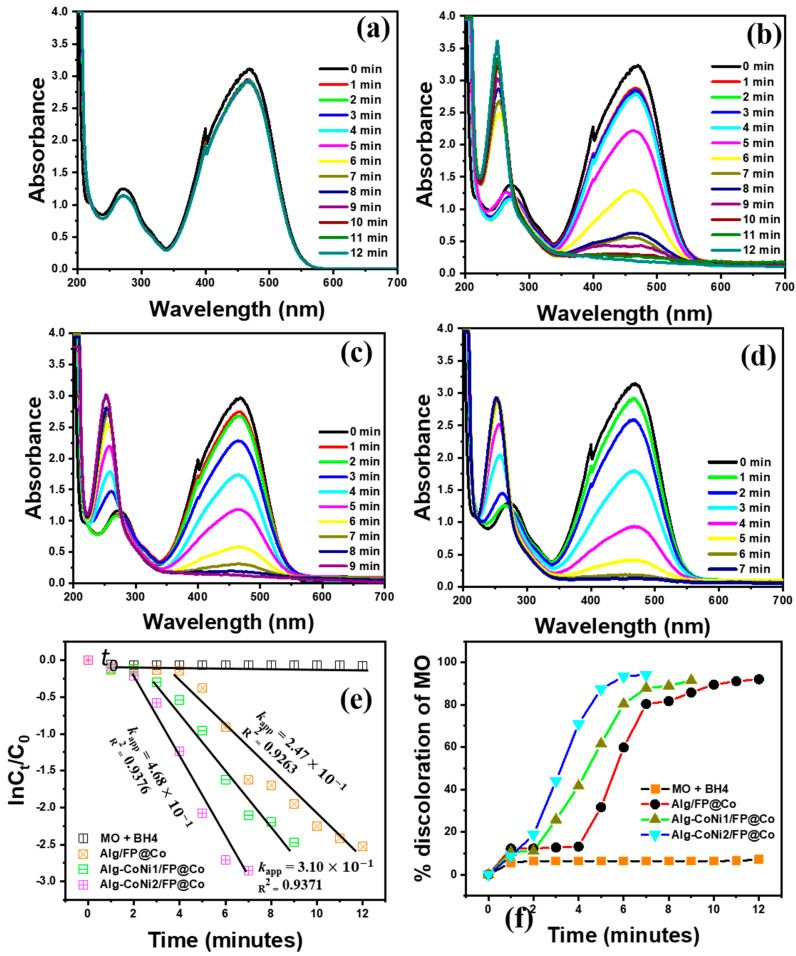
UV-Vis. absorbance spectra of MO dye discoloration in the presence of NaBH_4_ (**a**), and Alg/FP@Co (**b**), Alg-CoNi1/FP@Co (**c**), and Alg-CoNi2/FP@Co NPs (**d**), linear relationship of pseudo-1st order kinetics (**e**), and percent degradation of the MO dye (**f**).

**Figure 6:**
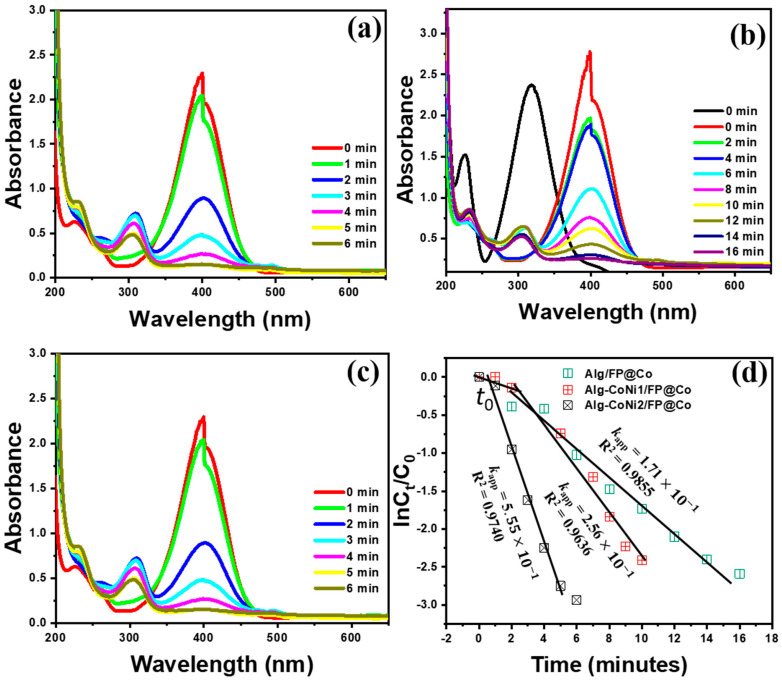
UV-Vis. absorbance spectra of 4NP reduction in the presence of NaBH_4_ using Alg/FP@Co (**a**), Alg-CoNi1/FP@Co (**b**), and Alg-CoNi2/FP@Co NPs (**c**), and linear relationship of pseudo-1st order kinetics (**d**).

Similar experiments were also conducted for MO dye discoloration for evaluating all the catalysts activity as discussed for CR dye degradation. As clear from the absorbance spectrum of Figure 5a, the NaBH_4_ has a negligible effect on the degradation of MO dye. Therefore, the catalyst was introduced along with NaBH_4_ for the MO dye degradation. Initially, we used Alg/FP@Co NPs (Figure 5b), where the MO dye decolorized in 12 min. After that, the effect of CoNi composite was studied on Alg/FP. The catalyst Alg-CoNi1/FP@Co NPs (Figure 5c) has a significant effect where it took 9 min for MO discoloration. Interestingly, an increased amount of CoNi composite in Alg/FP increased the rate of reaction. For instance, Alg-CoNi2/FP@Co NPs decolorized MO dye in 7 min (Figure 5d). During the degradation of MO dye with all catalysts, it was observed that MO dye appeared at λ_max_ 464 nm, which can react with NaBH_4_ to make their hydrazine product. The hydrazine product is oxidized after the addition of the respective catalyst and a new peak ascends at λ_max_ 251 nm, which is due to the formation of an amine functional group. The linear relationship lnC_t_/C_0_ vs. t (Figure 5e) indicates the superior catalyst activity of Alg-CoNi2/FP@Co NPs with *k*_app_ 4.68 × 10^−1^ min^−1^. An induction period *t*_0_ was observed in the degradation of MO dye. This time mainly encounters in catalysis reactions where the surface atoms organize themselves and expose their active sites such as faces, edges, and planes. Therefore, after this period the active sites become ready and the catalyst react very fast. Thus, induction period is very important for such a catalyst system. The percent degradation of MO dye with the respective catalyst indicated that approximately 7% of MO dye was degraded in 12 min by NaBH_4_ alone, while in the same time Alg/FP@Co NPs degraded 92% dye in the presence of NaBH_4_. Similarly, 91 and 94% efficiency was achieved with Alg-CoNi1/FP@Co and Alg-CoNi2/FP@Co NPs in 9 and 7 min, respectively (Figure 6f). This indicated that NaBH_4_ alone cannot change the reaction, while the catalyst brought an actual change in the reactant nature. The present work for CR, MO, and 4NP was compared with the literature data as shown in Table 4.

#### 3.3.3. Reduction of 4NP

4NP is considered a benchmark reaction for the evaluation of such a catalyst system. Therefore, all the catalyst was applied for the reduction of 4NP under a similar experimental procedure discussed above for CR and MO dyes degradation. 4NP is a toxic aromatic compound and strict restriction has been made on their use beyond the permissible limit, which was clariefied by the United State Enviornmental Protection Agency (U.S. EPA). This restriction is implemented on the use of nitrophenols because of their mutagenic and carcinogenic effect, as well as their adverse effect on lungs, kidneys, and CNS. 

It was studied in detail that neither borohydride nor catalyst alone reduced nitrophenol because of the high activation barrier requirements. However, both catalyst and NaBH_4_ together reduced the 4NP easily. As seen in the Figure 6a, Alg/FP@Co NPs can reduce 4NP to 4-aminophenol (4AmP) in 16 min, while Alg-CoNi1/FP@Co (Figure 6b) and Alg-CoNi2/FP@Co NPs (Figure 6c) took 10 and 6 min respectively, which further support the superior catalyst activity of Alg-CoNi2/FP@Co NPs. As depicted in all the absorbance spectra 4NP (318 nm) converted to 4-nitrophenolateanion having a λ_max_ value of 400 nm. Over time, the absorbance peak at 400 nm and their deep yellow color vanished with the rise of a new peak at 290 nm. This new peak is the indication of 4-AmP product [52,53]. Figure 6d exhibited a relationship of lnC_t_/C_0_ vs. time, which shows the highest rate constant value of 5.55 × 10^−1^ min^−1^ for Alg-CoNi2/FP@Co NPs. 

Based on the discoloration/reduction of azo dyes and nitrophenol it is inferred that CoNi composite played an important role in the reaction and facilitated the transfer of electrons for the degradation/reduction process. 

## 4. Conclusions

In the current study, various solid supports comprised of Alg/FP, Alg-CoNi1/FP, and Alg/CoNi2/FP were designed for the stabilization of zero-valent Co NPs. The as-synthesized catalysts were used for the inhibition of *B. subtilis*, and the result revealed that Alg-CoNi2/FP@Co NPs exhibited the highest activity by inhibiting the bacterium zone to 2.5 cm. Furthermore, these catalysts were used as a dip-catalyst for the degradation of CR and MO dyes and reduction of 4NP. It was revealed that increasing the amount of CoNi nanocomposite increased the bacterial inhibition as well as the rate of dyes degradation and reduction of 4NP. These results suggest that CoNi nanocomposite has a major role in the polymer network for the chemical and biological studies. The CoNi nanocomposite probably facilitates the movement of electrons for the degradation of dyes. The rate constant values were deduced from the pseudo-first-order kinetics. The highest rate was displayed by Alg-CoNi2/FP@Co NPs for MO dye, which was 4.68 × 10^−1^ min^−1^. Similarly, the *k*_app_ of Alg-CoNi2/FP@Co NPs against the degradation of CR dye is 3.63 × 10^−1^ min^−1^ and lowest shown by Alg/FP@Co NPs. Among these different catalysts, Alg-CoNi2/FP@Co NPs displayed the highest activity against both chemical and biological activities. 

## Figures and Tables

**Table 1:** Zone of inhibition of *B. subtilis* by all four catalysts.

Catalyst	Zone of Inhibition (cm)
Alg/FP	0.3
Alg/FP@Co NPs	1.0
Alg-CoNi1/FP@Co NPs	1.7
Alg-CoNi2/FP@Co NPs	2.5

**Table 2:** Kinetics parameters based on pseudo-1st order and % degradation of CR, MO dyes, and 4NP.

Targeted Pollutants	Catalyst	*k*_app_ (min^−1^)	R^2^	% Degradation
CR	Alg/FP@Co	1.27 × 10^−1^	0.9382	91.76
	Alg-CoNi1/FP@Co	1.46 × 10^−1^	0.9694	91.65
	Alg-CoNi2/FP@Co	3.63 × 10^−1^	0.9084	90.41
MO	Alg/FP@Co	2.47 × 10^−1^	0.9263	92.02
	Alg-CoNi1/FP@Co	3.10 × 10^−1^	0.9371	91.55
	Alg-CoNi2/FP@Co	4.68 × 10^−1^	0.9376	94.22
4NP	Alg/FP@Co	1.71 × 10^−1^	0.9855	92.50
	Alg-CoNi1/FP@Co	2.56 × 10^−1^	0.9636	91.06
	Alg-CoNi2/FP@Co	5.55 × 10^−1^	0.9740	94.69

**Table 3:** Effect of concentration, catalyst dosage, and reducing agent on the discoloration of CR dye using Alg-CoNi2/FP@Co NPs as a *dip*-catalyst.

Reaction Condition	Various Reaction Effect	Reaction Parameters	*k*_app_ (min^−1^)	R^2^	% Degradation
0.5 mL NaBH_4_ + 30 mg catalyst	Concentration	0.03 mM	3.63 × 10^−1^	0.9568	86.8
		0.05 mM	2.57 × 10^−1^	0.9139	91.65
		0.09 mM	3.63 × 10^−1^	0.9084	90.41
3 mL of 0.09 mM CR dye solution + 30 mg catalyst	NaBH_4_ (1 mM)	0.5 mL	3.63 × 10^−1^	0.9084	90.42
		1 mL	3.68 × 10^−1^	0.9076	89.91
		2 mL	6.98 × 10^−1^	0.9508	94.85
3 mL of 0.09 mM CR dye solution + 0.5 mL of 1 mM NaBH_4_ solution	Catalyst dosage	30 mg	3.68 × 10^−1^	0.9084	90.42
		60 mg	4.28 × 10^−1^	0.8897	90.42
		90 mg	5.97 × 10^−1^	0.9074	93.82

**Table 4:** Comparative literature data for CR, MO, and 4NP degradation with the present data.

Catalyst	Targeted Pollutant	Concentration (mg/L)	Amount of Catalyst (g/L)	% Discoloration	References
CA-PS-ZnO	CR	50.00	__	95.0	[47]
Ba/Alg/CMC/TiO_2_		30.00	1.20	95.0	[48]
Ba/Alg/CMC		30.00	1.20	58.0	[48]
Alg-CoNi2/FP@Co		48.72	8.44	90.4	Present work
cellulose/TiO_2_ monolith	MO	20.00	0.19	__	[49]
PtNPs@KWP		20.00	__	99.0	[50]
Alg-CoNi2/FP@Co		22.89	8.44	94.2	Present work
CelA_C2–F-5%	4NP	0.10	10.0	85.7	[51]
Alg-CoNi2/FP@Co		21.00	8.44	94.6	Present work

## Data Availability

N/A.
